# Mouse HFM1/Mer3 Is Required for Crossover Formation and Complete Synapsis of Homologous Chromosomes during Meiosis

**DOI:** 10.1371/journal.pgen.1003383

**Published:** 2013-03-21

**Authors:** Michel F. Guiraldelli, Craig Eyster, Joseph L. Wilkerson, Michael E. Dresser, Roberto J. Pezza

**Affiliations:** 1 Oklahoma Medical Research Foundation, Oklahoma City, Oklahoma, United States of America; 2 Department of Cell Biology, University of Oklahoma Health Science Center, Oklahoma City, Oklahoma, United States of America; Stowers Institute for Medical Research, United States of America

## Abstract

Faithful chromosome segregation during meiosis requires that homologous chromosomes associate and recombine. Chiasmata, the cytological manifestation of recombination, provide the physical link that holds the homologs together as a pair, facilitating their orientation on the spindle at meiosis I. Formation of most crossover (CO) events requires the assistance of a group of proteins collectively known as ZMM. HFM1/Mer3 is in this group of proteins and is required for normal progression of homologous recombination and proper synapsis between homologous chromosomes in a number of model organisms. Our work is the first study in mammals showing the *in vivo* function of mouse HFM1. Cytological observations suggest that initial steps of recombination are largely normal in a majority of *Hfm1^−/−^* spermatocytes. Intermediate and late stages of recombination appear aberrant, as chromosomal localization of MSH4 is altered and formation of MLH1foci is drastically reduced. In agreement, chiasma formation is reduced, and cells arrest with subsequent apoptosis at diakinesis. Our results indicate that deletion of *Hfm1* leads to the elimination of a major fraction but not all COs. Formation of chromosome axial elements and homologous pairing is apparently normal, and *Hfm1^−/−^* spermatocytes progress to the end of prophase I without apparent developmental delay or apoptosis. However, synapsis is altered with components of the central region of the synaptonemal complex frequently failing to extend the full length of the chromosome axes. We propose that initial steps of recombination are sufficient to support homology recognition, pairing, and initial chromosome synapsis and that HFM1 is required to form normal numbers of COs and to complete synapsis.

## Author Summary

Meiosis is a specialized type of cell division during which haploid gametes are generated to offset doubling of the chromosome number that occurs at fertilization. Prior to segregation in the first meiotic division, meiotic chromosomes pair and recombine. Chiasmata, products of recombination, provide the temporary physical link between the chromosomes of each pair. This association is required to prepare the chromosomes for segregation to the opposite poles of the spindle at the first meiotic division, so that ultimately each gamete receives only one copy of each chromosome. For this reason, errors in recombination are commonly linked to human infertility, spontaneous abortions, and severe aneuploid-based birth defects. We observed that the absence of HFM1/Mer3 results in deficient recombination and in incomplete meiosis-specific modification of chromosome structure. Our findings thus indicate that the mouse HFM1 protein is required for normal progression of important aspects of meiosis that act to insure that the correct numbers of chromosomes are transferred to the next generation.

## Introduction

Meiosis is a reductional type of cell division in which two rounds of chromosome segregation follow one round of DNA replication. This is a highly regulated and essential process to generate gametes containing the correct haploid chromosome complement. The first segregation, meiosis I, is reductional because homologous chromosomes, instead of sister chromatids, migrate to the opposite poles of the spindle. Recombination in meiosis I prophase prepares homologous chromosomes for segregation. Chiasmata, the cytological manifestation of recombination, provide physical links that hold the homologs in pairs and ensure the proper segregation of each chromosome of a pair to opposite poles of the spindle so that ultimately each gamete receives one copy of each chromosome [1], [2]. Mutations that reduce the level of recombination are invariably associated with increased errors in meiotic chromosome segregation [3], [4]. Examples of human diseases caused by aneuploidy include Down, Klinefelter, Edwards and Turner syndromes [5]. In order to explain the several causes of aneuploidy we need to identify and understand the mechanisms of homologous recombination that regulate proper number and positioning of COs in meiotic chromosomes.

The initial steps of homologous recombination involve introduction of double-strand breaks (DSBs) by the topoisomerase-like protein SPO11 [6], [7], [8], [9] followed by DNA end processing by exonucleases to generate 3′ single-stranded DNA tails [10], [11], [12]. Subsequently, RAD51 and DMC1 recombinases form filaments on the resected DNA tails and, together with ancillary proteins [13], [14], [15], catalyze the search for homologous sequences, resulting in repair of DSBs by promoting the invasion of intact double-stranded DNA by the single-stranded DNA ends (initial strand invasion) [15], [16], [17]. It is currently accepted that strand invasion intermediates proceed by one of two distinct pathways. They may dissociate after limited heteroduplex extension by DNA synthesis with subsequent re-joining of the broken chromosome by synthesis-dependent strand annealing to generate non-crossovers (NCO) [9], [18], [19]. Alternatively, initial strand invasion becomes stabilized by 3′ to 5′ unwinding of duplex DNA that promotes synthesis-mediated extension and gives rise to more stable single-end invasions [20], [21], [22] and double Holliday joins (dHJs) [23]. Intermediates in the second pathway complete repair to generate COs [18], [20], [21], [23], [24], [25]. Regulation of CO products is critical in meiosis but the players and mechanisms of control are poorly understood in higher eukaryotes. Two pathways have been proposed for the formation of COs in higher eukaryotes [26], [27], [28], [29], [30], [31], [32]. The majority of COs (90–95% in mouse) are processed by the ZMM group of proteins which include SYCP1 protein from the transverse filaments [33], [34], [35], [36], [37] of the synaptonemal complex, the ATP-dependent helicase Mer3 (HFM1 in mouse) [28], [38], [39], [40], the MSH4-MSH5 complex [34], [41], [42], [43], [44] and the MLH1-MLH3 heterodimer. This class of COs, termed class I, exhibits unique properties such as interference and homeostasis [45], [46], [47]. The second class of COs (Class II) depends on the structure specific endonuclease MUS81 that promotes the alternative MUS81-MMS4 pathway [48], [49].

Based on sequence homology with proteins known to be DNA helicases, mouse and human HFM1 and other Mer3 homologs are predicted to have seven motifs characteristic of the DExH box type of DNA/RNA helicases [40]. Mer3-deficient budding yeast strains carrying mutations in some of these motifs exhibit meiotic defects in DNA recombinational repair [50]. Biochemical evidence indicates that purified recombinant Mer3 from budding yeast works at an early step of recombination by stabilizing initial strand invasion catalyzed by RAD51 via DNA heteroduplex extension [51].

Mutants that inactivate HFM1/Mer3 orthologs in yeast, Arabidopsis, rice, *Coprinus cinereus* and *Sordaria macrospora* demonstrate the importance of Mer3 in the normal progression of several critical aspects of meiosis. Molecular analysis of intermediates of recombination in yeast Mer3 mutants reveals that DSBs are formed normally but that repair is delayed, single-end invasions are reduced (reaching a maximum of 15% of wild-type levels) and formation of meiotic recombination-dependent DNA synthesis is rare [21], [24]. Formation of later intermediates of recombination, dHJs, is delayed and severely reduced. This is accompanied by a 50–60% decrease in CO formation, and the remaining COs do not show interference [21], [24], [50], [52]. Similarly, genetic analysis of Arabidopsis Mer3 mutants shows that formation of COs is severely reduced (by 38%) but not eliminated, and that the remaining COs do not show interference [28], [38]. Notably, even though COs are severely affected in budding yeast, NCO intermediates are detected at normal levels in the absence of Mer3 [21], [24]. Cytological studies of Mer3 mutants suggest conserved function(s) of HFM1/Mer3 across all analyzed species. *Hfm1/mer3* mutants display severe deficiencies in chiasma formation, high levels of chromosome missegregation at meiosis I and partial or total infertility [21], [24], [28], [38], [39], [50], [52], [53], [54]. For budding yeast, Arabidopsis, rice, *C. Cinereus* and *S. macrospora* Mer3 mutants, development of axial elements is apparent normal and, except for *S. macrospora*, where homolog alignment is inefficient, the onset of chromosome pairing shows no differences with respect to wild-type [21], [28], [38], [39], [50], [52], [53], [54]. Notably, later in prophase I, synaptic defects are manifested in all *Hfm1/mer3* mutants. However, there is a wide variation in the extent of synapsis failure across species. Budding yeast and *C. cinereus* show the most severe synaptic phenotype, where the onset of synaptonemal complex formation is delayed [21], [24], [39]; in yeast, up to 90% of nuclei have separated homologous axes. Abnormal synapsis is also observed in *S. macrospora* where it persists among late pachytene cells and is accompanied by a high level of interwoven chromosomes, the latter being more severe after chromosome alignment [54]. Defects in synapsis are apparently moderate in Arabidopsis and rice, where EM and immunofluorescence analyses show only a partial asynapsis of chromosomes at pachytene [38], [53]. Results are controversial in Arabidopsis, however, in which one report describing the phenotype of four mutant alleles shows normal synaptonemal complex formation and no defect in synapsis [28]. These differences may reflect allelic differences. Nevertheless, failures in normal synaptonemal complex assembly across species indicate that the requirement for Mer3 in chromosome synapsis is evolutionarily conserved. Variability in the severity of synapsis defects and DNA repair abnormalities is reflected in the ability of Mer3 mutant meiocytes in different species to progress throughout prophase I. In yeast, arrest in meiotic progression results in dramatic reduction of spores and this strong arrest has been proposed to occur at late leptotene [21], [24], [50], [52]. In *S. macrospora* Mer3 mutants, the leptotene-pachytene transition is delayed, accompanied by defects in bouquet dynamics [54]. Initial stages of meiosis in both *C. cinereus* and Arabidopsis Mer3 mutants are apparently normal. However, in *C. cinereus* the majority of cells do not progress beyond meiosis I and became apoptotic [39]. In contrast, in Arabidopsis, one report shows that anomalous chromosome behavior first becomes apparent at diakinesis where all chromosomes are present as univalents which segregate randomly at metaphase I [28].

In this study we provide the first analysis of meiotic progression in HFM1/Mer3 null mice. We show that mutants of both sexes are infertile and that males show meiotic arrest at the end of prophase I. In a majority of *Hfm1^−/−^* spermatocytes the number of initial recombination events appears comparable to that observed in wild-type mice, but recombination is defective and the number of bivalents at diakinesis is reduced to ∼20%. These results are consistent with current models of recombination in which HFM1 participates in the major CO pathway. Although CO formation is severely defective in *Hfm1^−/−^* spermatocytes, chromosomes pair and initial synapsis is apparently normal. Our results suggest that early recombination transactions are sufficient for homologous recognition, pairing and initial synapsis. However, synapsis is incomplete for all but a small number of chromosomes in *Hfm1^−/−^* spermatocytes. We propose that completion of synapsis generally depends on an HFM1 activity that leads to the formation of COs.

## Results

### HFM1 is required for completion of meiosis

**Figure 1:**
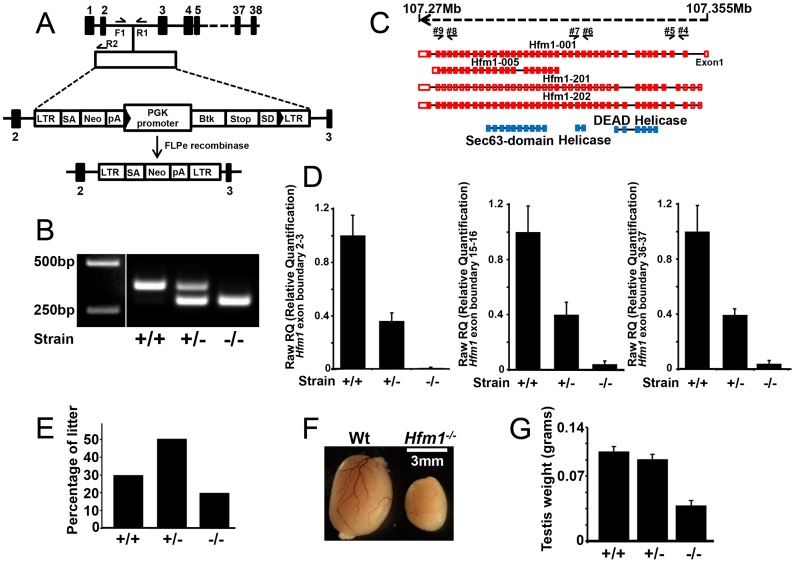
*Hfm1* gene target design and expression of *Hfm1* in mutant mice. (a) The mouse Hfm1 gene-targeting construct. A trapping cassette was inserted into intron 2 of the Hfm1gene. F1/R1 and F1/R2 represent primers used for genotyping wild-type and knockout mice, respectively. Exons are shown as numbered black bars. (b) Genotyping strategy for identification of *Hfm1^−/−^* mice. (c) Predicted alternative splicing variants of *Hfm1* (Hfm1 001, 005, 201 and 202). Exons are shown as red boxes and blue boxes represent portions of the HFM1 protein with recognized domains. Black arrows indicate the positions of primers used for RT-PCR analysis. (d) Transcript levels in testes of wild-type (+/+), heterozygous (+/−), and homozygous (−/−) mice measured by real time RT-PCR. (e) Percentage of litter obtained from *Hfm1* heterozygous couples. (f) *Hfm1^−/−^* males display significantly smaller testes than wild-type littermates. (g) Quantification of testis weight for wild-type, heterozygous and homozygous mice.

To understand the role of HFM1/Mer3 in mammals we generated *Hfm1^−/−^* mice. The gene trap vector utilized to disrupt *Hfm1* contains a viral long terminal repeat (LTR) followed by a splice acceptor sequence (SA) and a promoter-less selectable marker neomycin (Neo) with a polyadenylation signal (pA). The vector also contains the PGK promoter followed by the first exon of the Bruton's Tyrosine Kinase (Btk) gene upstream of a splice donor (SD). The Btk exon contains termination codons in all reading frames to prevent translation of downstream fusion transcripts (Figure 1a). *Hfm^+/−^* and *Flpe^+^* intercross results in the formation of *Hfm^−/−^* mice (Figure 1b–1g) in which no *Hfm1* RNA transcript is detected (Figure 1c and 1d). Two pairs of oligonucleotides specific for RNA transcript in three different regions of *Hfm1* were tested (exon boundaries 2–3, 15–16 and 36–37). RT-PCR analysis of *Hfm1^−/−^/Flp^+^* mice detected no *Hfm1* transcripts for any of the predicted alternative splicing forms of *Hfm1*(Figure 1c and 1d). Thus, we have generated mice that do not express any part of the HFM1 protein.

*Hfm1^−/−^* mice are viable. Out of the first 196 pups born, 39 (19.9%) are homozygous *Hfm1^−/−^*, a number that is close to a normal Mendelian distribution, and suggests that *Hfm1* is not an essential gene (Figure 1e). *Hfm1^−/−^* adult mice appear normal in all aspects except for reproductive tissues. *Hfm1^−/−^* males (0.044 g±0.005, n = 10) had significantly smaller testes than wild-type (0.1 g±0.0063, n = 8; P≤0.0001, t test) littermates (Figure 1f and 1g). This substantial reduction in size is similar to those observed following deletion of other meiosis-specific homologous recombination genes, including *Hop2*, *Dmc1 and Mlh1* [3], [55], [56].

**Figure 2:**
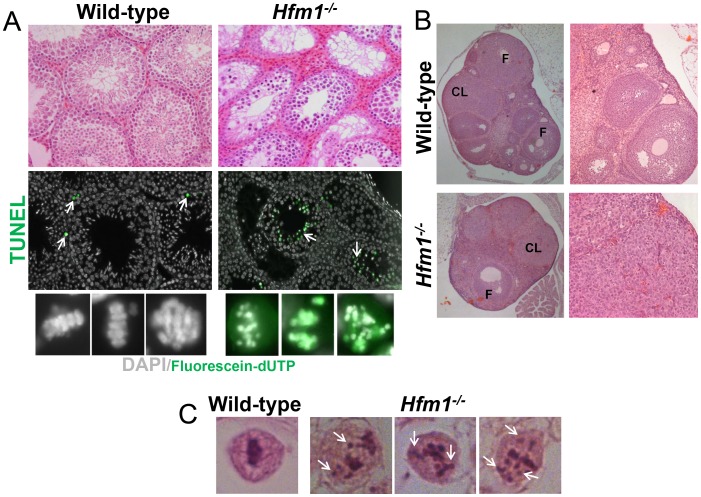
*Hfm1^−/−^* mice show profound defects in gametogenesis. (a) Histological sections of wild-type and *Hfm1^−/−^* testes. TUNEL assay detecting apoptotic cells is also shown (middle and lower rows). Arrows mark examples of cells after incorporation of Fluorescein-dUTP. The bottom row shows higher magnification images of diakinesis spermatocytes. (b) Histological sections of wild-type and *Hfm1^−/−^* ovaries. CL and F identify corpus lutea and follicles respectively. (c) Single spermatocytes showing a typical metaphase I plate arrangement of chromosomes in wild-type but lagging chromosomes scattered throughout the nucleus in *Hfm1^−/−^*.

Both male and female homozygous mutant mice were sterile due to severe blocks of spermatogenesis and oogenesis. Tissue analysis indicates that *Hfm1^−/−^* males develop testicular hypoplasia with hyperplasia of interstitial cells and lack spermatozoa. The number of seminiferous tubules is not reduced but the diameter is 30%–50% of normal. Primary spermatocytes represent the most advanced spermatogenic cells in the *Hfm1^−/−^* mice, indicating that spermatogenesis is blocked at diakinesis of meiosis I (Figure 2a, top row). TUNEL analysis for detection of apoptotic cells reveals that in contrast to wild-type, in which only occasional apoptotic cells are present, apoptosis of spermatocytes at diakinesis is common in seminiferous tubules of *Hfm1^−/−^* mice (Figure 2a, green cells after incorporation of Fluorescein-dUTP). We also analyzed hematoxylin-eosin histological sections of 45-days-old wild-type and *Hfm1^−/−^* ovaries. We note a significant reduction in ovary size, increase in stromal cells, reduced number of folicules and corpora lutea in the *Hfm1^−/−^* knockout mice. Nonetheless, we observed a small number of corpora lutea suggesting that ovulation could occur in the HFM1-deficient females, albeit infrequently (Figure 2b). Matings of heterozygous mice of both sexes produced litters of normal size but mating involving *Hfm1^−/−^* females were unsuccessful, suggesting that these females were infertile (results not shown).

In summary, tissue analysis suggests that spermatogenesis is apparently normal until the end of prophase I when spermatocytes arrest at diakinesis and indicates that prophase I checkpoints are not triggered in *Hfm1^−/−^* mice. Chromosomes appeared to be normally condensed at metaphase I in *Hfm1^−/−^* cells but, in contrast to wild-type cells, the chromosomes often failed to form a metaphase plate and remained scattered throughout the nucleus (Figure 2a (lower panel) and 2c).

### Cytological markers suggest that later stages of recombination are defective in *Hfm1^−/−^*

**Figure 3:**
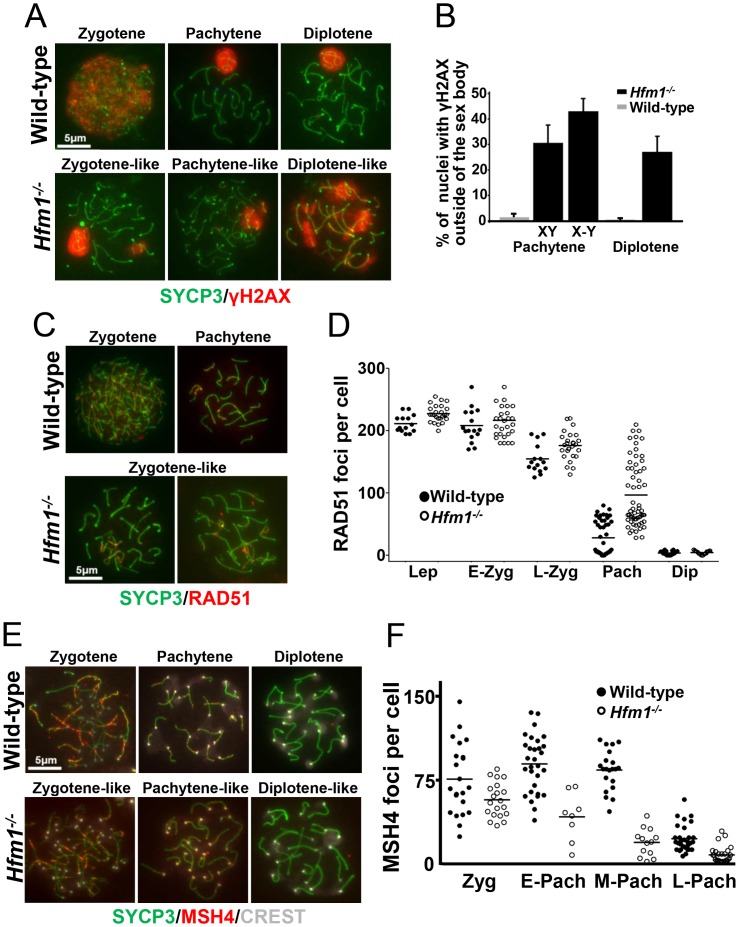
Abnormal accumulation of γH2AX, RAD51, and MSH4 foci in meiotic chromosomes of *Hfm1^−/−^* spermatocytes. (a) Chromosome spreads of wild-type and *Hfm1^−/−^* spermatocytes immunostained for SYCP3 and γH2AX. Magnification bar represents 5 µm. (b) Quantification of wild-type and *Hfm1^−/−^* spermatocytes showing additional γH2AX signal. (c) Representative spermatocytes of wild-type and *Hfm1^−/−^* mice at different stages of prophase I immunostained for SYCP3 and RAD51. Magnification bar represents 5 µm. (d) Quantification of the number of RAD51 foci per cell at the indicated stages. Lep, leptotene; E-Zyg, early zygotene; L-Zyg, late zygotene; Pach, pachytene and Dip, diplotene. Horizontal lines denote means. See Table 1 for summary of means, standard deviations, and results of statistical tests. (e) Spread nuclei of wild-type and *Hfm1^−/−^* spermatocytes immunostained for SYCP3 and MSH4. (f) Quantification of MSH4 foci per cell at the indicated stages. Horizontal lines denote means. See Table 1 for summary of means, standard deviations, and results of statistical tests.

As described in the Introduction, deletion of HFM1/Mer3 in a number of species results in abnormal progression of homologous recombination. We tested progression of recombination in mouse *Hfm1^−/−^* spermatocytes. DSB repair can be assayed indirectly by staining chromosomes for γH2AX [57], [58], which appears on chromatin near DSBs [59] then disappears with repair. Wild-type and *Hfm1^−/−^* pachytene spermatocytes were identified by typical morphological characteristics. Chromosomes look very compact and relatively stiff, at least 95% of the axial/lateral elements (SYCP3 signal) of homologous chromosomes are closely coaligned (synapsed), and sex chromosome axes appear in a typical pachytene conformation as previously defined by Dresser, et. al. [60]. In the case of γH2AX staining, for quantitation purposes, pachytene-like *Hfm1^−/−^* spermatocytes were subdivided into two categories by the state of X and Y chromosome pairing (XY = tightly associated and X-Y = loosely associated). We note that in wild-type cells at pachytene and diplotene, immunostaining with γH2AX marks only the sex body (n = 100). For a fraction of *Hfm1^−/−^* spermatocytes, however, some γH2AX persists not only in the sex body but also in one or several patches along autosomes in pachytene-like spermatocytes with either tightly (30.8%±7, n = 210) or loosely associated (43.2%±5.1, n = 120) X and Y chromosomes. Remnants of γH2AX can also be observed in a fraction of diplotene *Hfm1^−/−^* spermatocytes (27.4%±6.2, n = 240) (Figure 3a and 3b).

RAD51 forms cytologically-detectable complexes at sites of ongoing recombination [61], [62]. We determined the kinetics of formation and disapeareance of RAD51 foci on meiotic chromosomes of wild-type and *Hfm1^−/−^* mice. RAD51 foci decrease from late zygotene (154.6±21.6 foci per cell, n = 15) to pachytene (27.9±27.1 foci per cell, n = 55) in wild-type spermatocytes but were elevated in *Hfm1^−/−^* spermatocytes in late zygotene (176.1±22.2 foci per cell, n = 28, P≤0.001, t test) and persisted at particularly high levels in 31% of pachytene-like nuclei (96.9±41.2 foci per cell, n = 80, P≤0.001, t test) (Figure 3c and 3d and Table 1). Persistent RAD51 foci at pachytene, as previously described for yeast and *S. macrospora* Mer3 mutants [52], [54], may indicate delayed and/or inefficient DSB repair. However, it is possible that persistent RAD51 foci reflect the failure to remove RAD51 from dsDNA even though repair is complete.

**Table 1:** Number of recombination-associated foci in spermatocytes.

Protein	Stage	Genotype			
		Wild-type	N	*Hfm1^−/−^*	N
RAD51	Leptotene	208.3±25.1	16	227.6±12.9	32
	Early zygotene	205.2±21.8	16	221.6±38.6	27
	Late zygotene	154.6±21.6	15	176.1±22.2^a^	28
	Pachytene	27.9±27.1	55	96.9±41.2^a^	80
	Diplotene	4.6±2.7	50	5.4±2.1	45
MSH4	Zygotene	75.8±32.8	22	56.7±15.9^b^	20
	Early pachytene	84.1±18.2	30	41.1±22.1^a^	8
	Mid pachytene	81.7±26.0	21	17.5±12.6^a^	13
	Late pachytene	21.0±12.2	35	6.1±7.6^a^	40
MLH1	Pachytene	24.5±2.1	152	0.8±3.5^a^	100

a Significantly different from wild-type (p≤0.0001, t test).

b Significantly different from wild-type (p≤0.02, t test).

In sum, our results suggest that DSBs are formed essentially at wild-type levels in *Hfm1^−/^* ^−^ spermatocytes, and that for a major fraction of *Hfm1^−/^* ^−^ cells the numbers and kinetics of γH2AX and RAD51 immunosignals are similar to wild-type.

MSH4 is a member of the mammalian mismatch repair gene family whose members are involved in the control of meiotic recombination. Previous studies have shown that MSH4 is present in the nuclei of spermatocytes early in prophase I and that it forms discrete foci along meiotic chromosomes during the zygotene and pachytene stages of meiosis [43]. Compared to wild-type cells, *Hfm1^−/^* ^−^ spermatocytes showed a reduced number of MSH4 foci at zygotene (74.9% P≤0.041, t-test), early pachytene (48.8%, P≤0.0007, t test), mid pachytene (21.4%±, P≤0.0001, t test) and late pachytene (29%, P≤0.0001, t test) (Figure 3e and 3f and Table 1). Thus, HFM1 is required for accumulation of normal numbers of MSH4 foci.

**Figure 4:**
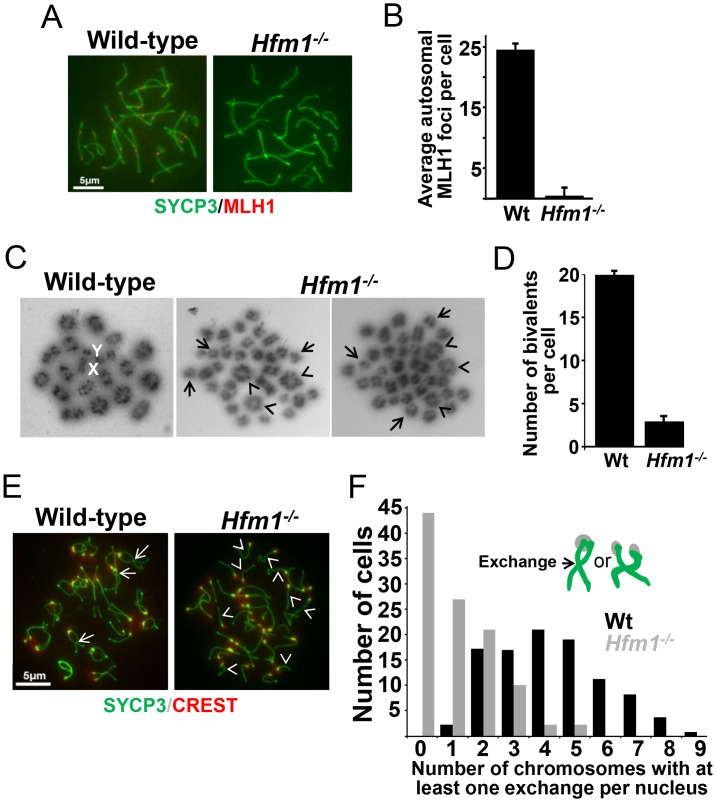
Reduced number of COs in HFM1-deficient spermatocytes. (a) Spread nuclei of wild-type and *Hfm1^−/−^* pachytene spermatocytes immunostained for SYCP3 and MLH1. Magnification bar represents 5 µm. (b) Quantification of autosomal MLH1 foci per pachytene cells. See Table 1 for summary of means, standard deviations, and results of statistical tests. (c) Metaphase spreads of wild-type and *Hfm1^−/−^* spermatocytes. Note the reduced number of bivalents (arrowheads) and increased number of univalents (tailed arrows) in *Hfm1^−/−^* cells. X and Y indicate the sex chromosomes. (d) Quantification (mean ± standard deviation) of metaphase bivalents per cell. (e) Representative diplotene wild-type and *Hfm1^−/−^* spermatocytes stained with anti-SYCP3 and CREST (a centromere marker) antibody. Note the increase in univalent chromosomes of knockout cells (arrowheads). Tailed arrows indicate examples of forming chiasmata in wild-type spermatocytes. Magnification bar represents 5 µm. (f) Quantification of cells in (e).

MLH1 mismatch repair protein is required for CO formation in mice. The accumulation of MLH1 foci along chromosome axes in early to mid pachytene is thought to occur at sites where COs are established [26], [63]. In agreement with published results, in wild-type cells we observed an average of 24.5±2.1 MLH1 foci per cell (n = 152) [63], [64], [65]. However, we observed only background signal in pachytene *Hfm1^−/−^* spermatocytes (0.8±3.5, n = 100) (Figure 4a and 4b and Table 1), suggesting a defect in the class I crossover pathway.

### Chiasma frequency is substantially reduced in *Hfm1^−/−^* spermatocytes

To confirm that deletion of HFM1 reduces CO formation, we analysed chiasma frequency in wild-type and *Hfm1^−/−^* spermatocytes. Chiasmata join homologs to make stable bivalents, so we first scored the number of bivalent chromosomes per cell in air-dried diakinesis spreads. We found an average of 20±0.5 (n = 100) bivalents per wild-type spermatocyte and only 3.7±2.2 (n = 100) bivalents per *Hfm1^−/−^* spermatocyte (Figure 4c and 4d). The dramatic reduction (81.3%, t test, P≤0.001) in the number of bivalents observed in *Hfm1^−/−^* cells strongly suggests a requirement for HFM1 in the formation of COs. As a second test, we measured the number of chromosomes at mid-diplotene with apparent mid-arm chiasmata (Figure 4e and 4f). We again observed a significant difference in the distribution of chiasmata per cell for wild-type (mode of 5 chiasmata per cell) compared to *Hfm1^−/−^* (mode of 0 chiasmata per cell) (P = 0.03, Wilcoxon rank-sum test). The reduction in the number of chiasmata in knockout spermatocytes indicates a requirement for HFM1 in the formation of the major fraction of COs in mice.

### HFM1 is required for normal levels of XY synapsis but not for sex body formation

**Figure 5:**
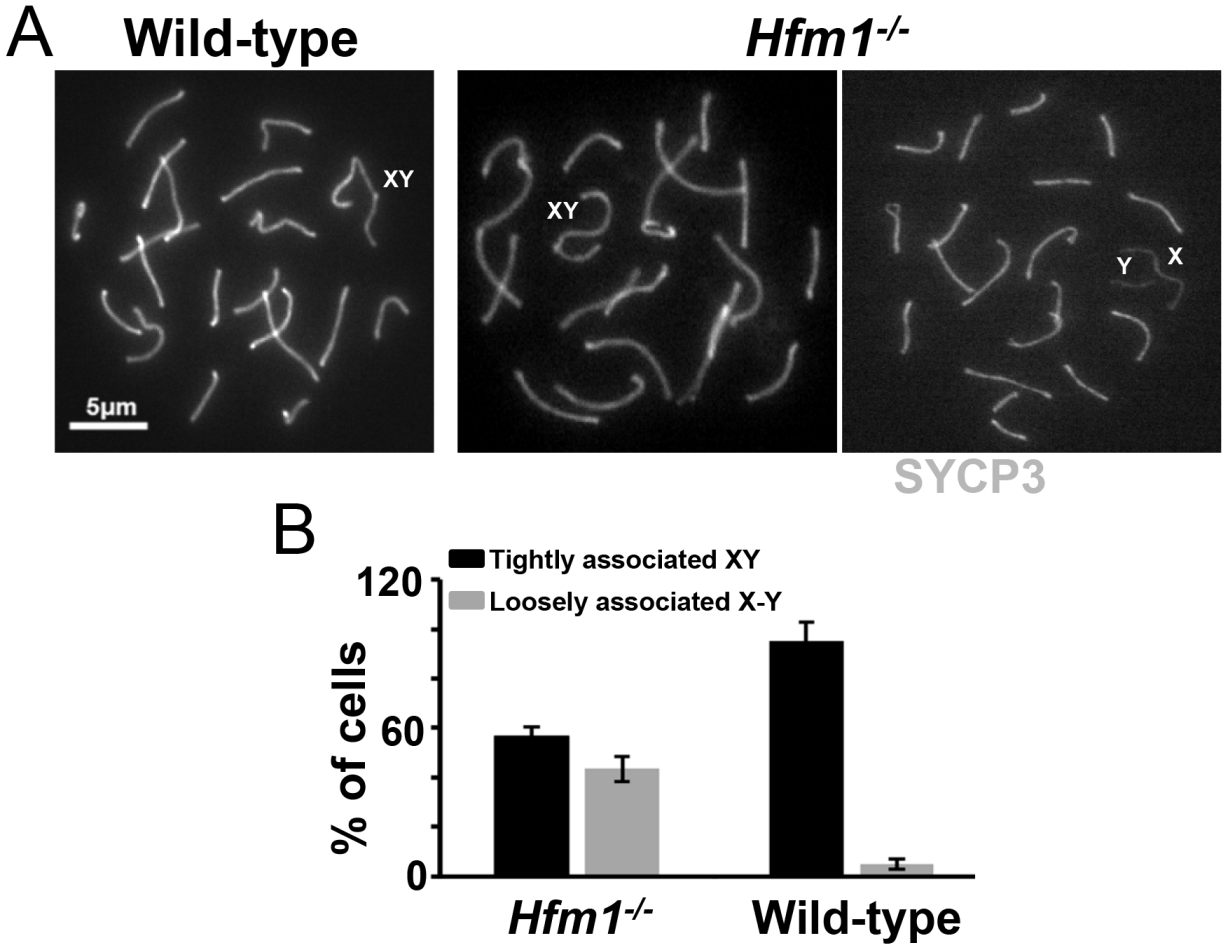
Effect of *Hfm1* deletion on sex chromosome association. (a) Representative pachytene wild-type and *Hfm1^−/−^* spermatocytes immunostained for SYCP3 showing tightly or loosely associated X and Y chromosomes. Magnification bar represents 5 µm. (b) Quantification of cells in (a). Bars represent standard deviation obtained from 4 wild-type and 3 *Hfm1^−/−^* mice.

In early pachytene spermatocytes, X and Y chromosome pairs normally form a short stretch of synaptonemal complex encompassing the small region of homology in the pseudo-autosomal region (PAR). Synapsis at PAR is very sensitive to reduced rates of recombination and perturbations in synapsis, and thus can be used to detect defects in these processes [65]. The sex body formed apparently normally in all pachytene and diplotene *Hfm1^−/−^* spermatocytes. However, the X and Y chromosome PAR regions are unsynapsed and separated in 43.5% of *Hfm1^−/−^* cells (n = 161) *versus* only 5% of wild-type cells (n = 110, P≤0.001 Fisher's exact test) (Figure 5a and 5b). It is possible that XY synapsis fails to initiate at wild-type frequencies. However, a later defect in synapsis (see below) suggests the alternative that XY association is lost prematurely.

### Homologous chromosomes pair but synapsis is frequently defective in *Hfm1^−/−^* spermatocytes

**Figure 6:**
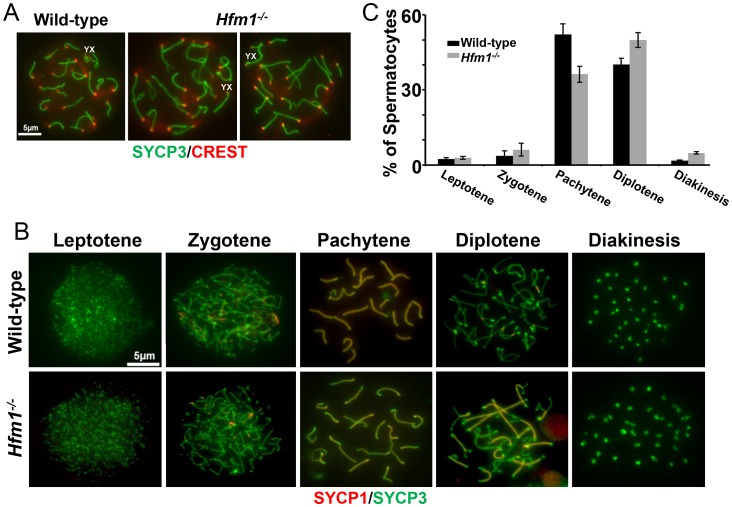
Meiotic prophase I progress in wild-type and *Hfm1^−/−^* mice. (a) Representative pachytene wild-type and *Hfm1^−/−^* spermatocytes stained with SYCP3 and CREST. Magnification bar represents 5 µm. (b) Co-immunostaining of SYCP3 and SYCP1 in wild-type and *Hfm1^−/−^* spermatocytes with no apparent synaptic defects at different stages of prophase I. Note that diakinesis is the last stage of spermatogenesis detected for *Hfm1^−/−^* cells. (c) Composition of spermatocyte population in wild-type and *Hfm1^−/−^* spermatocytes.

Given that meiotic recombination and synapsis (*i. e.*, synaptonemal complex formation) are codependent processes [66], we tested the requirement for HFM1 in synapsis by localizing synaptonemal complex proteins SYCP3 of the axial/lateral element and SYCP1 of the central element. Autosomal axes of similar lengths are found in coaligned pairs, indicating that development of the axial element of the synaptonemal complex and homologous chromosome pairing are normal in all *Hfm1^−/−^* spermatocytes. In addition, absence of splitting of the axes indicates that sister chromatid cohesion is not affected by the absence of HFM1 (21±0.7 centromeres for wild-type (n = 100) and 21±1.2 centromeres for *Hfm1^−/−^* (n = 100) spermatocytes at pachytene) (Figure 6a). Spermatocytes progress through meiotic prophase I in *Hfm1^−/−^* mice (Figure 6b and 6c). When individual stages of asynchronous populations of wild-type and *Hfm1^−/−^* spermatocytes from adult mice were compared, we observed an increase in the percentage of diplotene (wild-type, 40.1±2, n = 672; *Hfm1^−/−^*, 49.96±3, n = 686; P≤0.008, t test) and diakinesis (wild-type, 1.7±0.2, n = 20; *Hfm1^−/−^*, 4.81±0.5, n = 66; P≤0.0001 t test) and a decrease in pachytene (wild-type, 52.17±4.2, n = 614; *Hfm1^−/−^*, 36.27±3.2, n = 620; P≤0.0008, t test) in *Hfm1^−/−^* spermatocytes relative to wild-type. However, no significant differences were observed when the distribution of spermatocytes for the entire population of spermatocytes (from leptotene to diakinesis) from wild-type and *Hfm1^−/−^* were compared (Wilcoxan rank P = 0.3, one sided).

**Figure 7:**
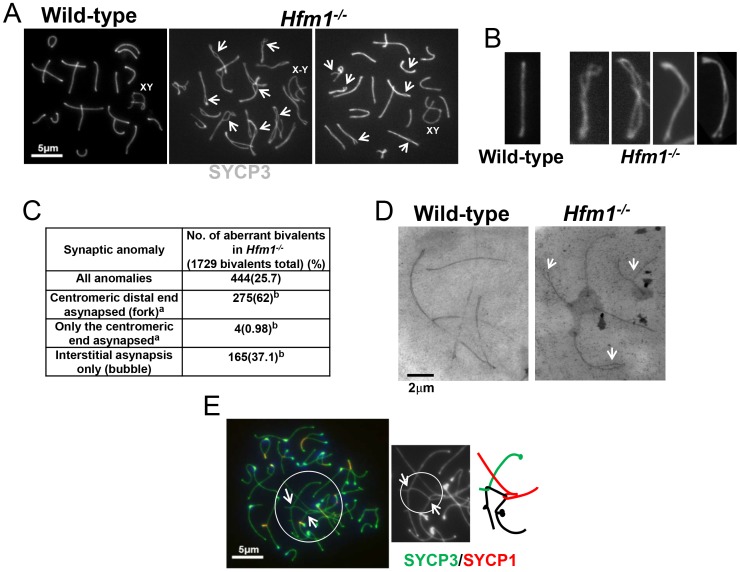
Homologous chromosomes pair normally and achieve initial synapsis in *Hfm1^−/−^* spermatocytes. (a) Immunostaining of SYCP3 in wild-type and *Hfm1^−/−^* spermatocytes. Examples of pachytene-like spermatocytes are shown. Arrows mark sites of synaptic defects. XY and X-Y indicate positions of tightly and loosely associated sex chromosomes, respectively. (b) Magnified chromosomes show details of synaptic defects. (c) Quantification of synaptic anomalies in meiotic chromosomes of *Hfm1^−/−^* spermatocytes. a - Irrespective of whether interstitial asynapsis was also present. b - % calculated with respect to total chromosomes displaying anomalies. (d) Electron microscopy of silver stained pachytene-like spermatocytes. Arrows mark sites of synaptic defects. (e) Example of tangled chromosomes in diplotene *Hfm1^−/−^* spermatocytes. Arrows indicate sites of entangled chromosome axes. Magnification bar represents 5 µm.

Notably, synaptic defects are evident early in pachytene spermatocytes (Figure 7a–7d). We analyzed the numbers and types of synaptic anomalies in pachytene where at least 95% of the axial/lateral elements (SYCP3 signal) of homologous chromosomes are closely coaligned (synapsed) and sex chromosome axes show the typical early pachytene configuration [60]. Pachytene-like spermatocytes with at least one abnormal synaptic conformation constituted 46.2% of all scored cells (n = 91) and 25.7% of total scored chromosomes showed some type of synaptic anomaly. Incompletely synapsed bivalents had one end unsynapsed, generally the centromere distal end (62% of the total chromosomes exhibiting anomalies) (Figure 7c). Thus, chromosome homologous recognition and pairing appears normal in *Hfm1^−/−^* spermatocytes but synapsis is frequently defective.

Chromosome interlocks, as visualized in the *mer3* mutant of *S. macrospora* [54], were not seen in *Hfm1^−/−^* spermatocytes but entangled chromosomes appeared in a fraction of *Hfm1^−/−^* diplotene nuclei (3.6%, n = 190; none were detected in wild-type, n = 320; P≤0.001) (Figure 7e). Whether this entangling occurs earlier but only becomes visible at diplotene, or develops only as chromosomes desynapse in diplotene, is not clear. It is possible that defects in resolving partial interlocks (e. g., those involving chromatin loops but not chromosome axes, which would be invisible in our assay) originated earlier in prophase and resulted in the synaptic discontinuities and entangling that we observed later in prophase.

**Figure 8:**
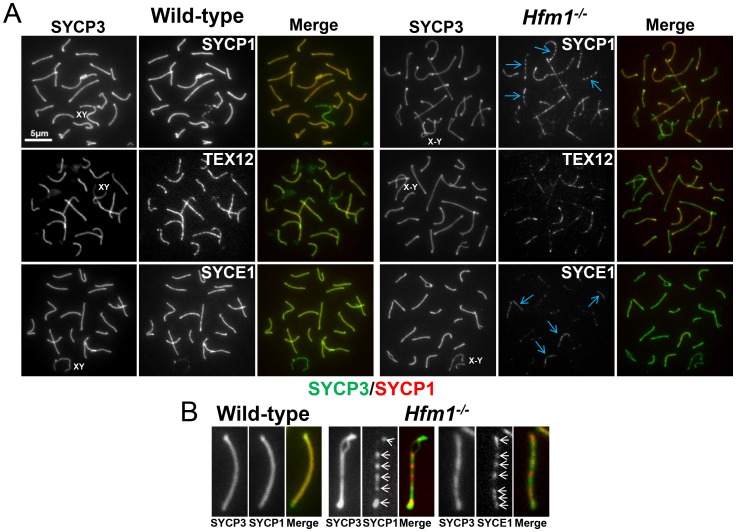
Synaptic defects in *Hfm1^−/−^* spermatocytes. (a) Defects in SYCP1, TEX12 and SYCE1 deposition on synaptonemal complex of *Hfm1^−/−^* pachytene-like spermatocytes. Wild-type is shown for comparison. XY and X-Y represents the sex chromosomes. Arrows indicate examples of chromosomes with patches of central element and transverse filament components of the synaptonemal complex. (b) Magnified chromosomes display details of patchy staining for SYCP1 and SYCE1 in *Hfm1^−/−^* spermatocytes. Arrows indicate SYCP1 and SYCE1.

**Figure 9:**
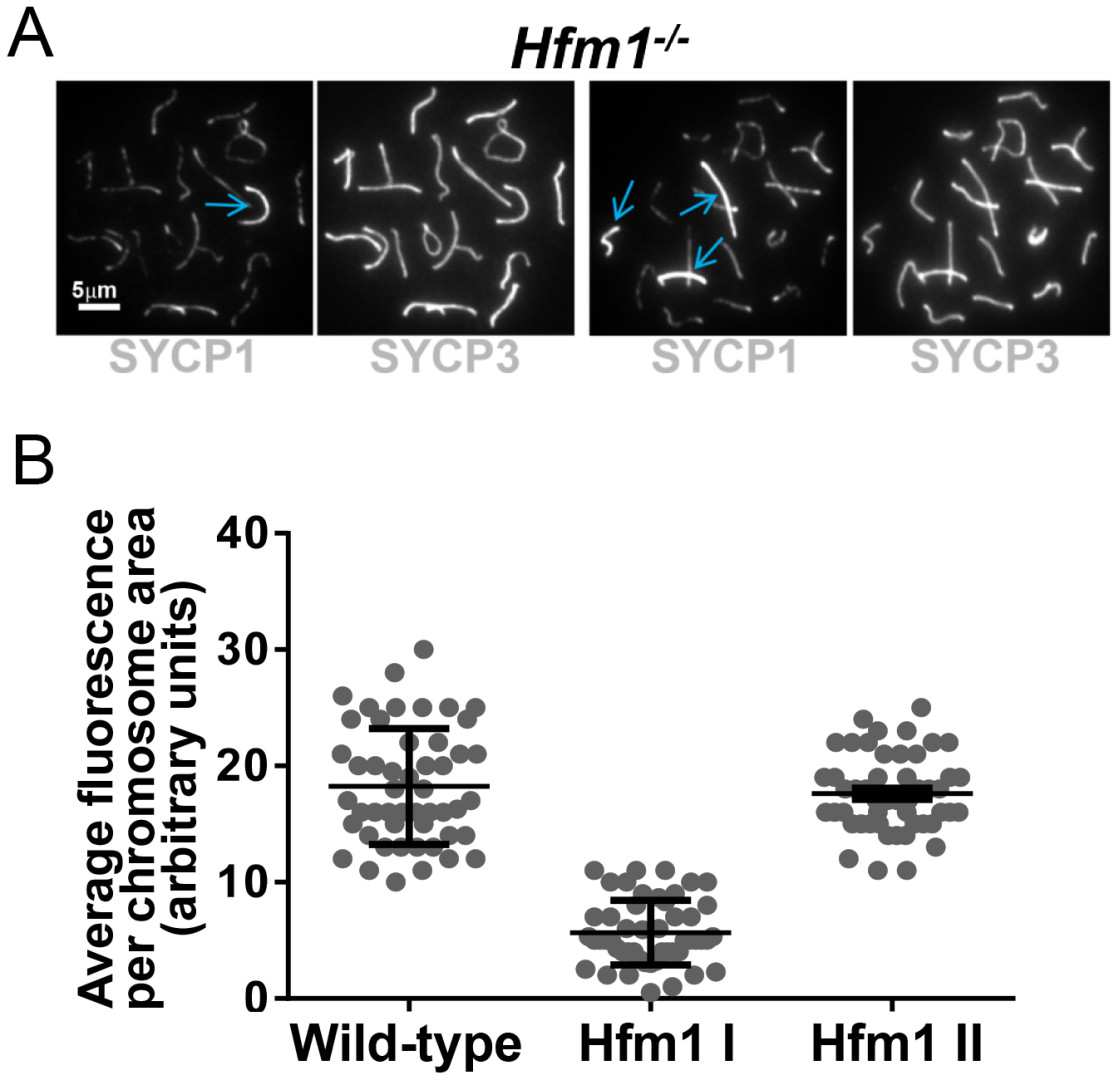
*Hfm1^−/−^* spermatocytes contain a small number of bivalents with wild-type levels of SYCP1 staining and uninterrupted synapsis. (a) *Hfm1^−/−^* nuclei with 1 (left) and 3 (right) bivalents displaying wild-type levels of SYCP1 staining. Arrows indicate chromosomes with wild-type like distribution and signal intensity for SYCP1. (b) Quantification of SYCP1 immunosignal for wild-type and *Hfm1^−/−^* spermatocytes. 50 chromosomes were randomly picked for wild-type and 100 chromosomes for *Hfm1^−/−^* spermatocytes. Hfm1 I and Hfm1 II represent chromosome populations from *Hfm1^−/−^* spermatocytes with and without reduced immunofluorescence with respect to wild-type. Plotted values were obtained using the Metamorph program to determine average intensity per chromosomal area, which was corrected for background fluorescence and normalized by the length of the chromosome.

A novel characteristic of chromosomes in *Hfm1^−/−^* spermatocytes is the substantially reduced immunostaining for SYCP1 (82.9%, n = 380) and components of the synaptonemal complex central element TEX12 (80%, n = 120) and SYCE1 (70%, n = 97) compared with wild-type pachytene cells, in which all chromosomes show strong, uninterrupted immunolabeling (scored chromosomes, n = 608) (Figure 8). In *Hfm1^−/−^* spermatocytes, SYCP1, SYCE1 and TEX12 localize in patches that in some areas are remarkably evenly spaced (Figure 8a and 8b; see [54]). Intriguingly, many *Hfm1^−/−^* spermatocytes contained a small number of bivalents with wild-type levels of SYCP1 staining and uninterrupted synapsis, even though the remainder of bivalents had uniformly reduced levels of SYCP1 and gaps in synapsis (60% *Hfm1^−/−^* spermatocytes with synapsed XY, n = 59, and 76% *Hfm1^−/−^* spermatocytes with unsynapsed XY, n = 67) (Figure 9a). The average intensity of fluorescent signal for SYCP1 from wild-type (18.24±0.70, n = 50 chromosomes) and from *Hfm1^−/−^* bivalents with reduced SYCP1 signal (Hfm1 I, 5.65±0.39, n = 50) are significantly different (P≤0.0001, t test). However, no significant difference was observed when wild-type was compared with the fraction of *Hfm1^−/−^* bivalents with higher levels of SYCP1 staining (Hfm1 II; 17.60±0.47, n = 50; P≤0.45, t test) (Figure 9b).

In summary, efficient homologous recognition and synaptic initiation occur in the absence of HFM1. However, in *Hfm1^−/−^* spermatocytes, (1) for all but a small fraction of chromosomes, synapsis is incomplete and characterized by subnormal amounts of central region components of the synaptonemal complex and by regions of complete asynapsis, (2) the X and Y chromosomes are frequently unsynapsed even though they colocalze in the sex body, suggesting premature desynapsis, though failure to initiate synapsis cannot be ruled out, and (3) chromosome entanglement becomes visible at diplotene.

## Discussion

### Recombination defects in HFM1/Mer3-deficient meiocytes

**Figure 10:**
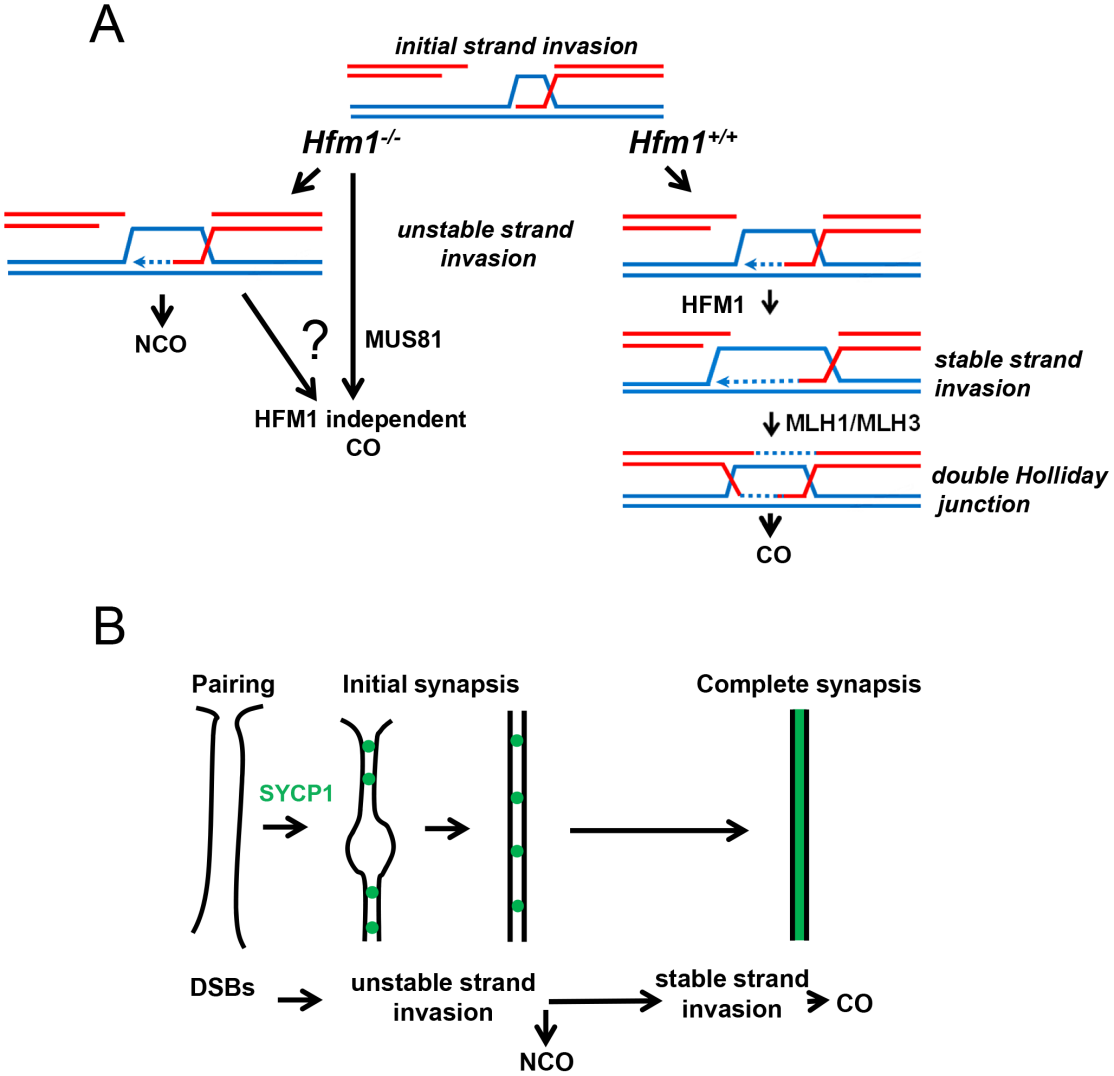
The meiotic role of mouse HFM1. The proposed model summarizes observations of this and previous studies. (a) HFM1 is required for normal progression of meiotic recombination. Deletion of HFM1 in spermatocytes leads to the absence of most but not all COs. (b) Initial stages of recombination are sufficient to support homologous chromosomes pairing and initial synapsis. However, in the absence of CO-specific intermediates synapsis cannot be completed along the full lengths of bivalents.

We observed that knocking out HFM1 in mouse spermatocytes results in a dramatic reduction of MLH1 foci and chiasma formation. These results indicate a recombination defect that inactivates the major pathway for CO formation. Similar observations have been made in mutants that inactivate Mer3 orthologs in budding yeast, Arabidopsis, rice, *C. cinereus* and *S. macrospora* [21], [24], [28], [38], [39], [50], [52], [53], [54]. Moreover, the physical analysis of intermediates of recombination performed in yeast Mer3 mutants revealed a dramatic decrease of single-end invasion and dHJ intermediates [21], which correlates with 50–60% disappearance of COs [21], [24], [50], [52]. Genetic analysis of Arabidopsis Mer3 mutants alleles also shows that formation of COs is severely reduced (by 38%) but not eliminated. In agreement, cytological analysis of Mer3 mutants in all studied species confirmed the severe deficiency in CO formation by showing a dramatic decrease in chiasma formation. This phenotype is in general correlated with cell cycle arrest or chromosome missegregation at meiosis I with partial or total infertility. CO formation in mouse *Hfm1^−/−^* spermatocytes is severely reduced but not eliminated (Figure 4). Interestingly, genetic analysis in budding yeast and Arabidopsis demonstrate that remnant COs do not show interference, therefore deletion of Mer3 led only to the elimination of interference-sensitive CO Class I [24], [28], [38], [50], [52]. By extension of these results and as previously proposed [26], [29], we speculate that at least two pathways for CO formation exist in mammals, the HFM1-dependent pathway which gives rise to interference-sensitive COs and another, presumably MUS81-dependent, pathway which is expected to produce a small number of randomly distributed COs [26] (Figure 10a).

An important observation from the molecular analysis of recombination intermediates performed in yeast is that while formation of COs is severely reduced, NCO intermediates are detected at normal levels in the absence of Mer3 [21], [24]. In this scenario it is attractive to think that in the major fraction of *Hfm1^−/−^* spermatocytes, DSBs are repaired as NCOs because most cells progress to diakinesis and show no immunosignal for markers of incompleted DNA repair such as γH2AX and RAD51. However, it is also possible that some DSBs are repaired as COs by using the sister chromatid as template.

An intriguing characteristic of the *Hfm1^−/−^* mutant spermatocytes is that the number of MSH4 foci in *Hfm1^−/−^* pachytene spermatocytes is substantially reduced with respect to wild-type, suggesting a defect in the middle stages of recombination. One possibility is that the deficiency of MSH4 may be explained by inefficient recruitment of this protein to chromosomes because HFM1 and/or other ZMM components are not loaded at recombination sites. However, other scenarios are possible. We also speculate that the less prominent decrease in MSH4 foci in zygotene may reflect an earlier HFM1-independent chromosome localization and perhaps function of MSH4. This may explain the more severe synaptic phenotype observed in MSH4 than in HFM1 mutants. Indeed, immunolocalization of Mer3 and Msh4 in yeast shows that only 50–70% of Mer3 foci co-localize with Mhs4 [67], and *Msh4^−/−^* and *Msh5^−/−^* [43] mice have a more severe meiotic phenotype than *Hfm1^−/−^*. However, further studies such as the analysis of Hfm1/Msh4 double mutants will be needed to test this possibility.

### HFM1/Mer3 is required for complete synapsis

Initial stages of meiotic chromosome restructuring are apparently not affected by HFM1/Mer3 deletion as in all tested species assembly of axial elements appears normal [21], [24], [28], [38], [39], [53], [54]. In addition, except for the case of *S. macrospora*, in which the onset of chromosome alignment is delayed [54], in all Mer3 mutants homologous recognition and chromosome pairing apparently proceed as in wild-type strains. Notably, another evolutionarily conserved characteristic of Mer3 is its requirement for normal synapsis. However, there is a wide variation in the extent of synapsis failure in Mer3 mutants in different species. Budding yeast and *C. cinereus* show the most severe phenotypes [21], [24], [39]. For example, Mer3 yeast cells stained with antibodies against Zip1 and Red1 show that up to 90% of cells have separated homologous axes [24], and EM analysis in *C. cinereus* mutants shows no formation of synaptonemal complex [39]. Abnormal synapsis is also observed in *S. macrospora* where asynapsed chromosomes persist among late pachytene cells and is accompanied by other chromosomal defects such as high levels of interwoven chromosomes [54]. We observed that in mouse *Hfm1^−/−^* spermatocytes, similar to rice and Arabidopsis Mer3 mutants, the defect in synapsis is apparently moderate. We found that a fraction of bivalents in pachytene have short lengths of widely separated axes and that components of the central region of the synaptonemal complex fail to extend along the entire lengths of most chromosome axes (Figure 7 and Figure 8).Thus, the requirement for HFM1/Mer3 to promote completed synapsis is conserved in mice.

A simple explanation for the largely normal pairing and early synapsis in mouse H*fm1^−/−^* spermatocytes is that initial recombination transactions, those occurring before HFM1 activity, are sufficient to establish recognition of homology between chromosome pairs (Figure 10b). The idea that earlier DNA transactions leading to NCOs as well as to COs promote initial stages of homologous chromosome interactions has been proposed previously [21], [54], and also finds support in double mutants of the budding yeast gene *sgs1* (a homolog of mammalian BLM) with *zip1*, *zip3* or *mer3*. In these mutants, interhomolog associations occur at many more “axial association” sites than the ones giving rise to COs and generate such uniform and close alignment of homologous axes that they appear “pseudosynapsed” [24], [68].

Although initial steps of recombination and chromosome pairing apparently are not affected by the absence of HFM1 and the concomitant reduction in the numbers of COs, incomplete synaptonemal complex formation is observed in *Hfm1^−/−^* spermatocytes. This suggests that completion of synapsis is dependent on later steps of recombination that lead to CO formation or to CO formation *per se*. Alternatively, the HFM1 contribution to completion of synapsis may be unrelated to its function in recombination.

The synaptic phenotypes of mouse mutants for proteins that apparently act before HFM1 (DMC1 and HOP2) and after HFM1 (MLH1 and MLH3) are consistent with the idea that HFM1 provides an “intermediate activity” in recombination that is required for completion of synapsis. In *Dmc1^−/−^* and *Hop2^−/−^* spermatocytes, homologous pairing and synapsis are absent [55], [56]. In contrast, in *Mlh1^−/−^* and *Mlh3^−/−^* spermatocytes, mature, complete synaptonemal complexes form [3], [69]. Our explanation of the synaptic phenotype in *Hfm1^−/−^* spermatocytes and the link to recombination may be extended to other model organisms with similar HFM1/Mer3-dependencies, as in Arabidopsis, rice and, to a lesser extent, *S. macrospora*. The more extensive synapsis defects observed in yeast and *C. cinereus* Mer3 mutants presumably indicate different dependencies in these organisms in the mechanisms directing and/or coordinating synaptonemal complex formation with recombination.

### Defects in *Hfm1^−/−^* spermatocytes do not cause developmental delay or cell apoptosis during prophase I

To avoid deleterious outcomes in recombination and synaptic defects, checkpoints survey meiotic errors and ultimately eliminate cells containing unresolved defects. In budding yeast and mice, among other species, meiocytes with defects in DSB repair and/or chromosome synapsis trigger meiotic arrest during prophase I [2], [70], [71], [72]. However, because recombination is interdependent with synapsis it has remained uncertain whether there is a distinct checkpoint that responds to defects in meiotic recombination or synapsis alone [73]. An intriguing characteristic of the HFM1 mutation in mouse spermatocytes is that despite abnormalities in recombination and synapsis, *Hfm1^−/−^* spermatocytes progress through prophase I with no apparent sign of checkpoint activation. For a major fraction of *Hfm1^−/−^* spermatocytes, we assume that most DSBs are repaired so that no intermediates remain that could trigger a DNA repair checkpoint. In this case, despite incomplete synaptonemal complex and partial defects, mutant spermatocytes are still able to satisfy the surveillance mechanisms. In this scenario, however, it is not clear to us why the fraction of *Hfm1^−/−^* spermatocytes that exhibit persistent γH2AX and RAD51 immunosignals do not show apparent prophase I delay or a cell response to an overly long delay/arrest such as apoptosis. A possible explanation is that persistent γH2AX and RAD51 foci are a consequence of inefficient removal of these proteins following abnormal (but completed) DNA repair. Alternatively, a low level of apoptosis during prophase I might not be detected by our assay. It also is possible that HFM1/Mer3 itself is required for a checkpoint, so that in the absence of Mer3 the checkpoint is inactivated. Regardless of the details, the requirements for HFM1/Mer3 for cell-cycle progression in mouse are different from those in budding yeast or *C.cinereus*, where Mer3 deletion results in mid prophase I arrest, or in *S. macrospora*, where Mer3 mutants show a delay in the leptotene-zygotene transition. These differences among species indicate that the biological functions that require HFM1/Mer3 and/or the responses to its absence have diverged along with other features of meiotic chromosome metabolism in these organisms.

## Materials and Methods

### Mice

Mice used in this study were wild-type (C57BL/6), transgenic Flpe B6.Cg-Tg (ACT FLPe) 9205Dym and HFM1 knockout. Experiments conformed to relevant regulatory standards and were approved by the IACUC (Institutional Animal Care and Use Committee).

### Generation of *Hfm1*-deficient mice

The mouse embryonic stem (ES) cell line Hfm1^Gt(ost347241)Lex^ containing a gene trap insertion in the second intron of the *Hfm1* gene (Figure 1a) was obtained from the Texas A&M Institute for Genomic Medicine (informatics.jax.org). The gene-trapping vector used to create this line, VICTR48, was designed to prevent translation of downstream fusion transcripts while maintaining the presence of the *Hfm1* transcript. To identify the exact insertion site within *Hfm1* intron 2, PCR reactions were performed using one primer hybridizing at the 5′ end of the gene trap vector and the complementary primer obtained by 3′ RACE PCR from the mouse embryonic stem cell clone *Hfm1* ^Gt(ost347241)Lex^. The PCR product was sequenced revealing that the insertion site was 1510 bp into intron 2. The Hfm1^Gt (ost347241) Lex^ embryonic stem cells were injected into C57BL/6 blastocysts to create chimeric mice, which were bred with C57BL/6 to generate the first progeny of *Hfm1^+/−^* heterozygous mice genotyped by PCR analysis (Figure 1b). Elimination of *Hfm1* transcript by removing the PGK/Btk/SD cassette was accomplished by crossing heterozygous *Hfm1* ^+/−^ mice with a mouse line expressing FLPe recombinase (B6.Cg-Tg (ACT FLPe) 9205Dym/J) (Figure 1a).

### Genotyping of mice by PCR

The genotyping was carried out by PCR using oligonucleotide #1 (F1) and #2 (R1) to amplify the wild-type allele (a 340 bp fragment) and primers #1(F1) and #3 (R2) (Table S1) to amplify the knockout allele (a 270 bp fragment). The cycling conditions were: 94°C 5 min; 94°C 30 sec, 58°C 45 sec, 72°C 60 sec for 35 cycles; 72°C 5 min. The presence of *Flpe* was assessed by genotyping using the primers (#10 and #11) under the same PCR conditions.

### Real-time RT–PCR

Total RNA was isolated from adult testis with the RNeasy Mini Kit (Qiagen). 2.0 µg of RNA was oligo dT primed and reverse-transcribed with the High capacity RNA-to-cDNA kit (Applied Biosystems). Three different exon boundaries of *Hfm1* were amplified using Power Syber Green PCR Master Mix (Applied biosystems) with the following primers: #4 and #5, #6 and #7 and #8 and #9, respectively. The cycling conditions were: 95°C 10 min; 95°C 10 sec, 60°C 30 sec, for 40 cycles. The melt curve was of 95°C 10 sec and 65°C to 95°C performed in increments of 0.5°C.

### Histological analysis

Testes and ovaries for histological examination were removed and fixed overnight in 10% neutral-buffered formalin (Sigma). Serial sections from either testes or ovaries were positioned on microscope slides and analyzed using either hematoxylin and eosin staining or a TUNEL assay (Roche).

### Cytology

We employed established experimental approaches for the visualization of chromosomes in chromosome surface spreads [74]. Incubations with primary antibodies were carried out for 12 h at 4°C in 1× PBS plus BSA 2%. To detect SYCP1 and SYCP3 we used polyclonal rabbit antibody raised against mouse SYCP1 at 1∶200 dilution (Novus Biologicals) and polyclonal mouse antibody raised against mouse SYCP3 at 1∶300 dilution (Abcam). Centromeres were detected using the human centromere protein antibody (CREST, Antibody Incorporated) at 1∶50 dilution. Other primary antibodies used in this study were as follows: monoclonal mouse antibody raised against mouse γH2AX at 1∶500 dilution (Millipore), polyclonal rabbit antibody raised against mouse RAD51 at 1∶200 dilution (Santa Cruz Biotechnology), polyclonal rabbit antibody raised against mouse MSH4 at 1∶200 dilution (AbCam), monoclonal mouse antibody raised against mouse MLH1 at 1∶50 dilution (BD pharmingen), polyclonal rabbit antibody raised against SYCE1 at 1∶200 dilution (kindly provided by Ch. Hoog) and polyclonal rabbit antibody raised against TEX12 at 1∶200 dilution (kindly provided by Ch. Hoog). Following three washes in 1× PBS, slides were incubated for 1 h at room temperature with secondary antibodies. A combination of Fluorescein isothiocyanate (FITC)-conjugated goat anti-rabbit IgG (Jackson laboratories) with Rhodamine-conjugated goat anti-mouse IgG and Cy5-conjugated goat anti-human IgG each diluted 1∶450 were used for simultaneous triple immunolabeling. Slides were subsequently counterstained for 3 min with 2 µg/ml DAPI containing Vectashield mounting solution (Vector Laboratories) and sealed with nail varnish. For RAD51, MSH4 and MLH1 focus counts, nuclei were staged using the extent of SYCP3 immunolabeling and synapsis as markers for meiotic prophase progression. Only foci that co-localized with the chromosome axes were counted. We use Axiovision SE 64 (Carl Zeiss, inc.) for imaging acquisition and processing. Statistical tests were as described in the text and table legends.

Spermatocyte chromosome spreads for electron microscopy analysis was performed as previously described [60].

## Supporting Information

Table S1 Summary of the oligonucleotide sequences used in this work.
(DOCX)
