# Supplementary figures and images for: Mouse HFM1/Mer3 Is Required for Crossover Formation and Complete Synapsis of Homologous Chromosomes during Meiosis
Source: PLoS Genet. 2013 Mar 21;9(3):e1003383. doi: 10.1371/journal.pgen.1003383 (PMC3605105; doi:10.1371/journal.pgen.1003383)

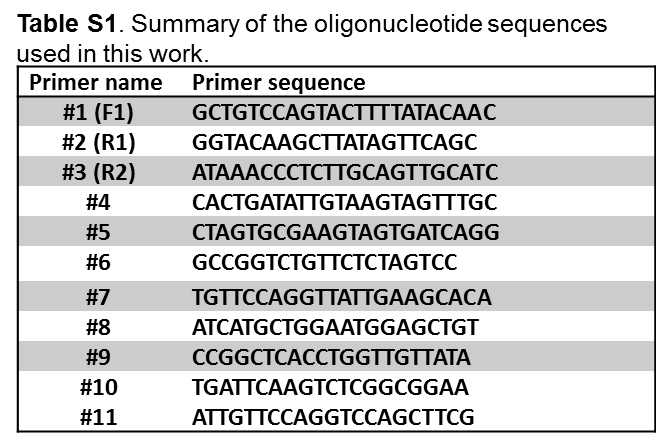
y

Supplement: Table S1 — Summary of the oligonucleotide sequences used in this work. (DOCX) [file pgen.1003383.s001.docx]
